# Defining and managing patients with non‐ST‐elevation myocardial infarction: Sorting through type 1 vs other types

**DOI:** 10.1002/clc.23308

**Published:** 2020-01-10

**Authors:** Marc Cohen, Gautam Visveswaran

**Affiliations:** ^1^ Division of Cardiology, Department of Medicine Newark Beth Israel Medical Center and Rutgers‐New Jersey Medical School Newark New Jersey USA

**Keywords:** acute coronary syndrome, anticoagulant, antiplatelet, myocardial infarction, non‐ST‐elevation myocardial infarction, revascularization, type 1 myocardial infarction, type 2 myocardial infarction

## Abstract

Advances in cardiovascular (CV) imaging, redefined electrocardiogram criteria, and high‐sensitivity CV biomarker assays have enabled more differentiated etiological classification of myocardial infarction (MI). Type 1 MI has a different underlying pathophysiology than type 2 through type 5 MI; type 1 MI is characterized primarily by intracoronary atherothrombosis and the other types by a variety of mechanisms, which can occur with or without an atherosclerotic component. In type 2 MI, there is evidence of myocardial oxygen supply‐demand imbalance unrelated to acute coronary atherothrombosis. Types 1 and 2 MI are spontaneous events, while type 4 and type 5 are procedure‐related; type 3 MI is identified only after death. Most type 1 and type 2 MI present as non‐ST‐elevation MI (NSTEMI), although both types can also present as ST‐elevation MI. Because of their different underlying etiologies, type 1 and type 2 NSTEMI have different presentation and prognosis and should be managed differently. In this article, we discuss the epidemiology, prognosis, and management of NSTEMI occurring in the setting of underlying type 1 or type 2 pathophysiology. Most NSTEMI (65%–90%) are type 1 MI. Patients with type 2 MI have multiple comorbidities and causes of in‐hospital mortality among these patients are not always CV‐related. It is important to distinguish between type 1 and type 2 NSTEMI early in the clinical course to allow for the use of the most appropriate treatments that will provide the greatest benefit for these patients.

## 1 INTRODUCTION

Acute coronary syndrome (ACS) includes a spectrum spanning unstable angina, non ST‐elevation myocardial infarction (NSTEMI), and ST‐elevation myocardial infarction (STEMI).^1^, ^2^, ^3^

Typically, ACS results from an abrupt total (STEMI and some NSTEMI) or subtotal (NSTEMI only) interruption of coronary artery blood flow, and therefore oxygen supply, to cardiac tissues.^1^, ^4^ This occurs as a result of coronary artery occlusion following atherosclerotic plaque disruption, where the rupture or erosion of an atherosclerotic plaque leads to the formation of an intraluminal thrombus in one or more coronary arteries.^1^, ^3^ Myocardial infarction (MI) can also result from distal thrombotic embolization.^1^, ^5^ Evaluation of the clinical presentation and determining the underlying pathophysiology of an MI are crucial for the development of an appropriate management plan.^1^, ^5^

STEMI is usually characterized by severe and/or total coronary flow obstruction with transmural ischemia, which predisposes to myocardial necrosis and pump dysfunction.^6^ The pathogenesis of NSTEMI differs from that of STEMI in that it usually results from a flow‐limiting coronary stenosis with resultant downstream myocardial ischemia.^1^, ^3^, ^4^ Total coronary artery occlusion is present in approximately one‐quarter of patients with NSTEMI.^4^ STEMI and NSTEMI require different approaches to their acute and long‐term management.^1^, ^2^, ^3^ In this article, we focus on the epidemiology, prognosis, and management of NSTEMI according to its underlying pathophysiology.

## 2 CLASSIFICATION OF MI

Clinically, MI is defined by the presence of acute myocardial injury, as detected by abnormal cardiac biomarkers (eg, cardiac troponins [cTn]) presenting with symptoms of myocardial ischemia with an abnormal electrocardiogram (ECG), imaging, or angiographic findings.^5^

### 2.1 Diagnosis of NSTEMI

The diagnosis of NSTEMI is covered in extensive detail elsewhere, including European and US clinical practice guidelines.^1^, ^3^ In summary, in NSTEMI, a 12‐lead ECG may show a depressed ST‐segment or T‐wave insertion, whereas in STEMI, an ECG shows persistent (>20 minutes) ST‐segment elevation or new left bundle‐branch block.^1^, ^2^, ^3^ Cardiac troponin testing, in combination with an ECG, has become an essential tool for accurately diagnosing MI and is mandatory for patients showing characteristics of an NSTEMI on ECG.^1^, ^3^ The Cardiac troponin test enables distinction between NSTEMI and unstable angina and therefore is an important aid in risk stratification and treatment decisions.^3^

Cardiac troponin is a specific cardiac structural protein associated with myocyte injury of any type.^7^, ^8^ Although elevated blood Cardiac troponin is not specific to acute coronary events, Cardiac troponin testing is highly sensitive in detecting small amounts of myocardial necrosis.^7^ In a study examining the utility of high‐sensitivity Cardiac troponin assays, more high‐risk patients presenting to the emergency department (ED) with unspecified chest pain were identified and admitted to the hospital than when conventional Cardiac troponin assays were used.^9^ This improved triage was associated with a reduction in major adverse cardiac events among patients directly discharged from the ED.^9^ The corresponding increase among patients who were admitted to hospital reflected the higher risk among this population.^9^

NSTEMI is characterized by Cardiac troponin values that follow a specific pattern, comprising an acute rise followed by a gradual fall, which is consistent with the patient's clinical presentation and ECG changes.^1^, ^3^

### 2.2 MI subtypes

**Figure 1:**
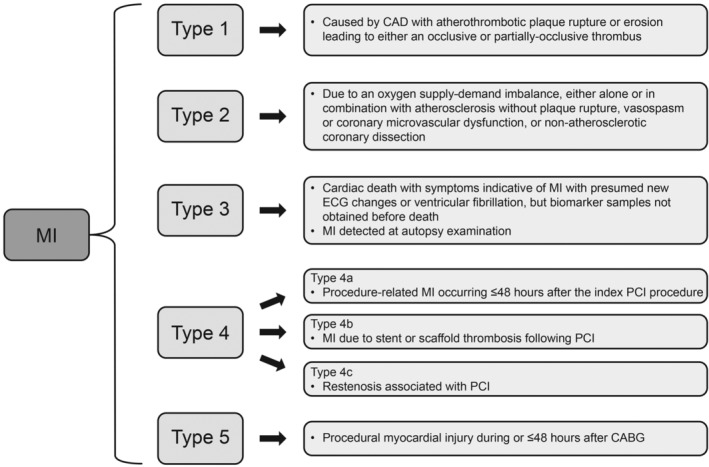
Classification of myocardial infarction based on the fourth universal definition.^5^ CABG, coronary artery bypass grafting; CAD, coronary artery disease; ECG, electrocardiogram; MI, myocardial infarction; PCI, percutaneous coronary intervention

In the past 20 years, the accuracy of detecting MI has improved, and more detailed examination of underlying pathophysiology has been possible due to advances in cardiovascular (CV) imaging, redefined ECG criteria, and availability of high‐sensitivity Cardiac troponin assays.^5^, ^10^ On the basis of this improved diagnosis of MI, the joint European Society of Cardiology/American College of Cardiology Foundation/American Heart Association/World Heart Foundation (ESC/ACCF/AHA/WHF) Task Force for the Redefinition of MI subdivided MI into five main categories according to etiology, with their most recent criteria for each MI type published in 2018 (Figure 1).^5^ Types 1 and 2 are spontaneous etiologies of MI, while type 3 is by definition fatal and type 4 and type 5 are procedure‐related.^5^ This review will focus on type 1 and type 2 MI.

Criteria for both type 1 and type 2 MI are a rise and/or fall in Cardiac troponin with ≥1 value higher than the 99th percentile of the upper reference limit and at least one of the following: symptoms of acute myocardial ischemia, new ischemic ECG changes, development of pathological Q waves, and imaging evidence consistent with ischemic etiology.^5^ Type 1 MI is characterized by atherothrombotic coronary artery disease (CAD) with an occlusive or non‐occlusive coronary thrombus, identified by angiography or autopsy.^5^ Type 2 MI differs from type 1 MI in that acute atherothrombotic plaque disruption is absent and there is evidence of an oxygen supply‐demand imbalance unrelated to acute coronary atherothrombosis, either alone or in combination with atherosclerosis, vasospasm or coronary microvascular dysfunction, or nonatherosclerotic coronary dissection.^5^

## 3 EPIDEMIOLOGY

### 3.1 Non‐ST‐elevation MI

The incidence of NSTEMI relative to that of STEMI has increased since the 1980s.^11^, ^12^, ^13^, ^14^ This increase in NSTEMI is likely the result of advances in medical care and technology that have facilitated the care of older patients with multiple comorbidities and, in part, the availability of high‐sensitivity Cardiac troponin assays for diagnosis of myocardial injury.^12^, ^14^ NSTEMI currently accounts for 60%–70% of MI hospitalizations.^11^, ^12^, ^13^, ^14^ About two‐thirds of those diagnosed with NSTEMI are men, although the proportion of women presenting with NSTEMI has increased in recent years.^11^, ^13^

NSTEMI is more common than STEMI, although the proportion of patients with STEMI is higher for type 1 MI than type 2.^15^, ^16^, ^17^, ^18^ A meta‐analysis of observational studies comparing type 1 and type 2 MI showed that 70.0% of the 2683 patients with type 2 MI were diagnosed with NSTEMI compared with 44.1% of 23 189 patients with type 1 MI.^19^ A prospective study of consecutive patients meeting the definition of MI found that 96.7% of the 144 patients with type 2 MI had NSTEMI, compared with 67.3% of the 397 patients with type 1 MI.^18^ However, it should be noted that a modified definition of type 2 NSTEMI was used that included clinical criteria relating to underlying conditions causing oxygen supply‐demand imbalance.^18^

### 3.2 Prevalence of type 1 and type 2 MI in NSTEMI

Results from a number of studies suggest that between 65% and 90% of NSTEMI events are pathophysiologically type 1 MI, depending on the clinical setting and the diagnostic criteria used.^16^, ^18^, ^20^ For example, in one prospective study of 541 hospitalized patients, 406 (73.4%) were diagnosed with NSTEMI; of these, 267 patients (65.8%) had type 1 NSTEMI and 139 (34.2%) had type 2 NSTEMI.^18^ A single‐center retrospective study of 1039 patients with a discharge diagnosis of NSTEMI found that 775 (74.6%) patients had type 1 NSTEMI and 264 (25.4%) had type 2 NSTEMI; none had type 4 or 5 MI.^20^ In contrast, in a larger prospective analysis of 19 763 hospitalizations for acute MI, the proportion of type 1 NSTEMI appeared to be even greater.^16^ Of the 13 211 patients who did not have ST‐segment elevation, approximately 90% had a diagnosis consistent with type 1 NSTEMI and only 10% with type 2 NSTEMI.^16^

Epidemiological studies may have underestimated the occurrence of type 2 NSTEMI because of the classification criteria used, as well as the clinical settings in which patients were identified.^16^, ^21^ Studies of patients admitted to a cardiac care unit or medical intensive care unit (ICU) are likely to include smaller proportions of patients with type 2 MI.^16^, ^22^ In one analysis of patients hospitalized with MI, 45.1% of patients with type 2 MI were admitted to a department other than a coronary care unit, compared with only 17.4% of those with type 1 MI.^18^ An analysis of 1112 patients presenting to an ED who had serial Cardiac troponin measurements and did not have STEMI found that among the 256 patients with NSTEMI, a type 2 MI was more common than type 1 MI (74.2% vs 25.8%).^23^ Because patients with type 2 MI tend to be older and have more comorbidities than those with type 1 MI, they may have already been admitted to general medicine wards because of another underlying illness and are not necessarily admitted to the ICU.^16^, ^22^ One study found that in 19% of a total of 446 744 hospital admissions with a diagnosis of acute MI, it was diagnosed as a comorbidity.^24^ Among these patients, the primary diagnoses leading to hospital admission included atherosclerotic CV disease, heart failure, hip fracture, and chronic obstructive pulmonary disease.^24^ While MI type was not determined, the authors suggested that these patients were likely to have had type 2 MI.^24^

In addition to differences in prevalence between clinical settings, type 2 MI is often incorrectly classified as type 1 MI because it was not recognized as a separate condition by the International Classification of Diseases (ICD) coding system until October 2017.^19^, ^25^, ^26^ An analysis of patients diagnosed with type 1 NSTEMI according to ICD‐9 and ICD‐10 codes between January 2015 and September 2017 found that 281 of 945 (29.7%) had type 2 MI and therefore required a different management strategy from those with type 1 MI.^26^

Forty‐five percent of patients diagnosed with a type 2 MI were reported to have no significant CAD, compared with only 12% of patients with type 1 MI.^18^ Underlying conditions that may predispose individuals to oxygen supply‐demand imbalances, and thus type 2 MI, include hypotension, sepsis, anemia, tachyarrhythmia, respiratory failure, and hypertension.^18^, ^22^ In addition to CV differences, a number of studies have shown that patients who have a type 2 MI are usually older, more often women, and more likely to have a history of comorbidities such as heart failure, arrhythmias, chronic obstructive pulmonary disease, or renal impairment.^16^, ^18^, ^19^, ^20^, ^27^, ^28^, ^29^

### 3.3 Prognosis following type 1 and type 2 NSTEMI

Among patients with NSTEMI of any type, in‐hospital and 30‐day mortality rates of between 5.2% and 13.1% and between 7.6% and 17.0%, respectively, have been reported.^11^, ^13^, ^14^ There has been some improvement in NSTEMI mortality rates over time, for example, from an in‐hospital mortality rate of 7.1% in 1994 to 5.2% in 2006,^11^ and from a 30‐day mortality rate of 10.0% in 1999 to 7.6% in 2008.^13^ Over the longer term, a 1‐year post‐discharge mortality rate following NSTEMI of 18.7% was reported for a US cohort in 2005, a decrease from 27.6% in 1999,^14^ with similar decreases observed in other studies.^30^, ^31^

The presence of comorbidities such as heart failure, atrial fibrillation, diabetes, impaired renal function, and older age influenced the likelihood of survival in the overall NSTEMI population.^14^, ^32^ Patients with type 2 NSTEMI are more likely to have these characteristics than those with type 1 NSTEMI, although they are less likely to have typical CAD risk factors.^20^, ^33^, ^34^

There are limited prognostic data for type 1 vs type 2 NSTEMI; however, in a single‐center retrospective study of 1039 patients with NSTEMI, those with type 2 vs type 1 MI had significantly higher in‐hospital mortality (17.4% vs 4.7%), 30‐day mortality (11.9% vs 2.2%), and 1‐year mortality (34.9% vs 12.4%).^20^ In‐hospital mortality was more likely to have a non‐CV cause among patients with type 2 MI than those with type 1 MI, even after adjustment for baseline characteristics, including comorbidities (adjusted odds ratio: 6.47; 95% confidence interval: 1.74–23.99).^20^

### 3.4 Management of type 1 and type 2 NSTEMI

**Figure 2:**
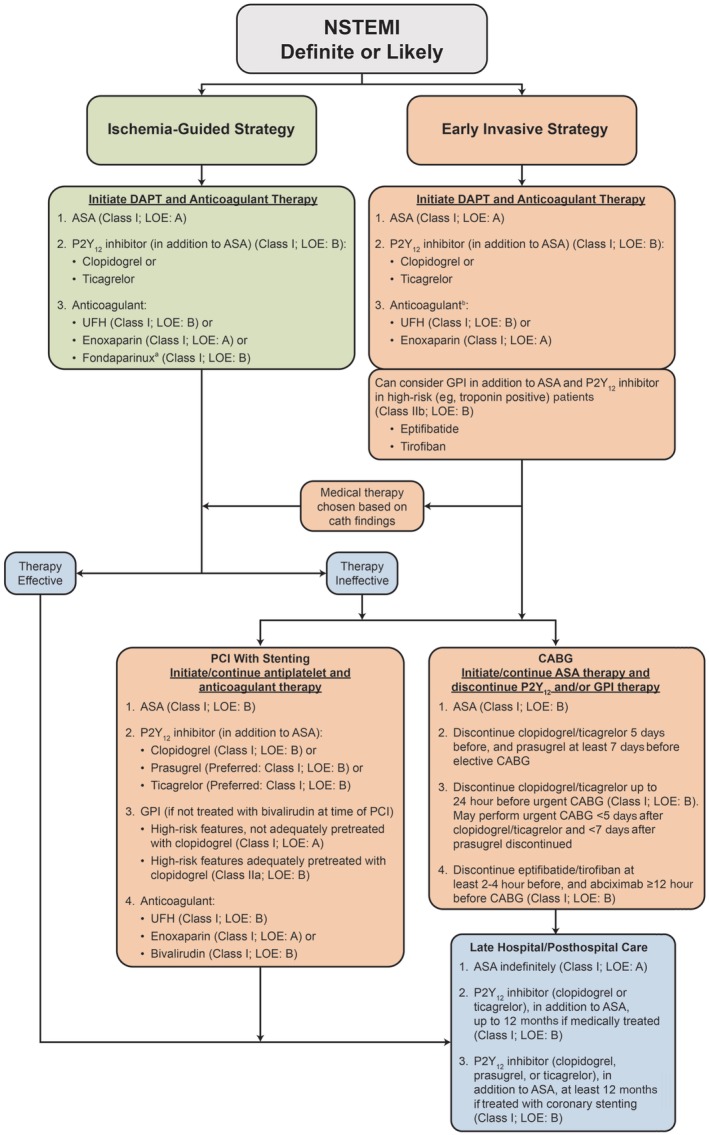
Algorithm for the management of non‐ST‐elevation myocardial infarction. Adapted with permission from Amsterdam et al^1^ ^a^Patients treated with fondaparinux should also receive an anticoagulant that inhibits factor IIa at the time of PCI to reduce the risk of catheter thrombosis. ^b^Bivalirudin (class I, LOE B) is also recommended for patients undergoing an early invasive strategy but is only used in the catheterization laboratory. Fondaparinux (class I, LOE B) is not routinely recommended; patients treated with fondaparinux should also receive an anticoagulant that inhibits factor IIa at the time of PCI to reduce the risk of catheter thrombosis. ASA, aspirin; CABG, coronary artery bypass graft; cath, cardiac catheterization; d, days; DAPT, dual antiplatelet therapy; GPI, glycoprotein IIb/IIIa inhibitor; h, hours; LOE, level of evidence; mo, months; NSTEMI, non‐ST‐elevation myocardial infarction; PCI, percutaneous coronary intervention; pts, patients; UFH, unfractionated heparin

All NSTEMI events should be diagnosed and managed in accordance with current treatment guidelines, with the aim of providing immediate relief of ischemia and preventing recurrent MI and/or death during the early hospitalization period, followed by long‐term prevention of secondary ischemic events.^1^, ^3^ After initial examination, stabilization, and risk assessment, an ischemia‐guided medical management strategy or an early invasive (angiography within 24 hours) strategy should be selected on the basis of individual patients' characteristics and risk level (Figure 2).^1^, ^3^

Initial NSTEMI management should proceed according to treatment guidelines,^1^, ^3^ regardless of suspected MI type. However, current guidelines do not differentiate between MI types in their recommendations and are more suited to patients with type 1 MI characteristics, including atherothrombotic plaque disruption, than those with other MI types.

#### 3.4.1 Initial assessment

Risk categorization tools such as the Global Registry of Acute Cardiac Events (GRACE) risk score and the Thrombolysis in Myocardial Infarction (TIMI) risk score can be utilized to assess both the acute and long‐term likelihood of a further ischemic event following an NSTEMI.^35^, ^36^ Ischemic risk scores have been found to be superior to clinical assessment alone for this purpose.^3^ The results of these risk assessments can thus be useful in patient management.^1^ Assessment of acute risk guides initial evaluation and selection of care facility, such as a coronary care unit, and the choice of appropriate pharmacotherapy, and guides decision‐making regarding invasive revascularization procedures.^3^

The TIMI and GRACE risk scores do not differentiate between type 1 and type 2 MI; however, patients with type 2 MI could be expected to have higher risk scores based on characteristics included in these risk scores such as older age, comorbidities, and prior history of MI.^35^, ^36^ An analysis comparing the characteristics of patients with type 1 and type 2 MI found that those with type 2 MI had a significantly higher mean GRACE score.^34^

#### 3.4.2 Antiplatelet therapy

Antiplatelet therapy is an essential first‐line component of guideline‐recommended type 1 NSTEMI treatment, to inhibit platelet activation and thus reduce acute ischemic complications and prevent further atherothrombotic events.^1^, ^3^ Dual antiplatelet therapy, consisting of a P2Y_12_ inhibitor plus aspirin, is indicated for patients who are initially treated with either an early invasive or medical management strategy.^1^, ^2^, ^37^, ^38^ Dual antiplatelet therapy guidelines support the use of the P2Y_12_ inhibitor ticagrelor rather than clopidogrel in type 1 NSTEMI, regardless of whether early revascularization is performed, based on the results of the Platelet Inhibition and Patient Outcomes (PLATO) study.^37^, ^38^, ^39^

Following successful recovery from the acute phase of an NSTEMI, patients remain at an increased risk of subsequent CV events and premature death.^40^, ^41^, ^42^, ^43^ Current NSTEMI guidelines therefore recommend that dual antiplatelet therapy with a P2Y_12_ inhibitor plus aspirin be continued for ≥12 months after the index date, after an assessment of the individual patient's ischemic and bleeding risk.^1^, ^3^, ^37^, ^38^

Recommendations for antiplatelet therapy are more applicable to patients with type 1 NSTEMI, as intracoronary thrombosis is absent in patients with type 2 MI.^5^ Furthermore, there is a lack of data on the use of dual antiplatelet therapy in patients with type 2 NSTEMI from either randomized controlled trials or observational studies.^44^

#### 3.4.3 Anticoagulant therapy

As with antiplatelet therapy, recommendations for anticoagulant treatment apply primarily to patients with type 1 MI because of the lack of atherothrombotic plaque rupture in type 2 MI. Administration of anticoagulants during the initial treatment of type 1 NSTEMI is effective in reducing ischemic events, and the combination of an anticoagulant and dual antiplatelet therapy during the acute phase is more effective for this than either treatment alone.^3^ Treatment with an anticoagulant during the acute phase is therefore recommended for all patients with a type 1 NSTEMI, regardless of the initial management strategy.^1^, ^3^ Unlike dual antiplatelet therapy, discontinuation of anticoagulants beyond the acute phase, for example, after percutaneous coronary intervention (PCI), should be considered in type 1 NSTEMI unless there is a compelling reason to continue.^1^, ^3^ For information on the management of patients who have another condition prompting continuation of anticoagulation, please refer to recent studies such as PIONEER AF‐PCI, RE‐DUAL PCI, and AUGUSTUS.^45^, ^46^, ^47^

#### 3.4.4 Revascularization

When considering revascularization in patients presenting with NSTEMI, the risks of morbidity and mortality associated with the procedure should be weighed against its benefits in terms of short‐ and long‐term prognosis, symptom relief, quality of life, and duration of hospital stay.^3^ Patients with type 2 NSTEMI are less likely than those with type 1 NSTEMI to undergo revascularization,^20^ and its benefit in type 2 NSTEMI has yet to be established.^26^ In addition to absence of intracoronary thrombosis, almost half of the patients with type 2 MI do not have significant underlying CAD.^5^, ^18^ Therefore, guideline recommendations for revascularization in patients with NSTEMI may be more relevant to type 1 MI. Early identification of atherothrombotic plaque disruption as the cause of the event is critical in triaging appropriate patients with type 1 NSTEMI to the cardiac catheterization lab and to distinguish it from type 2 NSTEMI.

The indication for an invasive approach, the timing of revascularization (immediate, early, or delayed), and the selection of the revascularization approach (ie, PCI or coronary artery bypass grafting [CABG]) in type 1 NSTEMI depend on a number of factors. These include the risk of a subsequent ischemic event, bleeding risk, and the clinical setting, for example, whether the patient has been admitted to a PCI center or will need to be transferred to a suitable facility.^3^ In general, guidelines recommend that in the case of multivessel CAD, the choice of CABG over multivessel PCI should be guided by the extent and complexity of the disease influenced by medical comorbidities.^1^

Large randomized clinical trials have shown that both the P2Y_12_ inhibitors prasugrel (in patients undergoing PCI) and ticagrelor (in patients managed with or without revascularization) significantly decrease the risks of CV death, MI, and stroke compared with clopidogrel.^48^, ^49^ Furthermore, in the PLATO trial, compared with clopidogrel, ticagrelor reduced ischemic events and total mortality in patients with type 1 NSTEMI or unstable angina who either underwent revascularization or who were medically managed.^39^ Guidelines for the management of type 1 NSTEMI therefore recommend ticagrelor in preference to clopidogrel in patients who undergo PCI or who are treated with medical therapy alone, and they recommend prasugrel over clopidogrel in patients who undergo PCI, except those who are aged ≥75 years, have a body weight of <60 kg, are at increased risk of intracranial hemorrhage, or have a history of stroke or transient ischemic attack.^37^

#### 3.4.5 Managing type 1 vs type 2 NSTEMI

**Figure 3:**
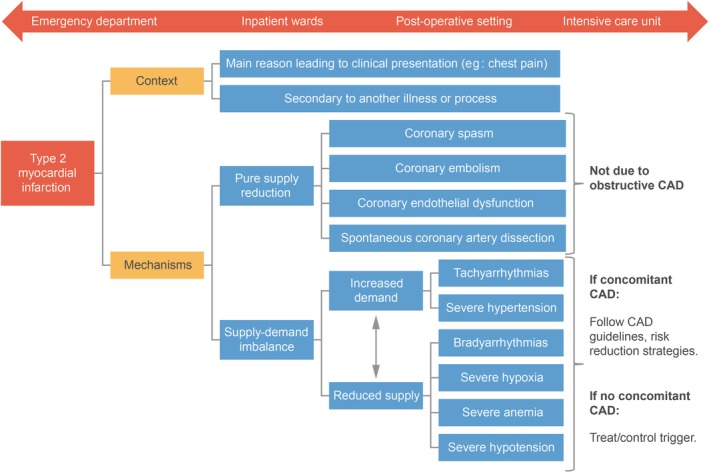
A proposed framework for management of type 2 myocardial infarction. Adapted with permission from Januzzi and Sandoval.^50^ CAD, coronary artery disease

Type 2 NSTEMI is often classified under a general NSTEMI diagnosis or misclassified as type 1, despite its presentation and underlying pathophysiology being different from those of type 1 NSTEMI.^26^ It is essential that NSTEMI be classified as type 1 or type 2, based on clinical presentation, Cardiac troponin, ECG, and imaging findings, so that it can be managed appropriately.^5^ Type 2 MI has heterogeneous underlying causes, often atypical features, a lack of a clear precipitating cause of the oxygen supply‐demand imbalance in some patients (eg, absence of discernible CAD), and does not usually involve atherothrombotic plaque disruption.^5^ As a result, current NSTEMI guidelines may be challenging—or in some cases inappropriate—to apply in patients who are experiencing a type 2 NSTEMI, and no formal guidelines for type 2 NSTEMI are available.^21^ Studies have found that compared with patients with type 1 NSTEMI, patients with type 2 NSTEMI were less likely to undergo catheterization and revascularization procedures or receive recommended secondary prevention medications at discharge, and they had higher mortality rates.^20^, ^26^ There is therefore a need for evidence‐based diagnosis and management guidelines for NSTEMI that acknowledge the complexities of type 2 MI.^16^, ^20^, ^21^, ^22^, ^28^ Consequently, a phenotype‐based approach to type 2 MI diagnosis and management across different clinical settings has been proposed (Figure 3).^50^ This approach considers both the context and multiple possible underlying mechanisms of type 2 MI and recommends treatment of the cause of the oxygen supply‐demand imbalance for each patient.^50^

## 4 SUMMARY AND CONCLUSIONS

Type 1 and type 2 MI have different etiologies. Type 1 is characterized primarily by CAD with atherosclerotic plaque rupture or erosion that leads to formation of an occlusive or partially occlusive thrombus, whereas the key feature of a type 2 MI is an oxygen supply‐demand imbalance, which can occur with or without an atherosclerotic component.^5^ Both type 1 and type 2 MI can present as NSTEMI or STEMI, although NSTEMI is more common for both types.^16^, ^17^, ^18^ Most NSTEMI pathophysiologically are consistent with type 1 MI, but the relative proportions of type 1 NSTEMI and type 2 NSTEMI vary between clinical settings.^16^, ^18^, ^19^, ^20^, ^27^, ^29^

Prognosis, in terms of short‐ and longer‐term mortality risks following NSTEMI, has improved in recent years, with fewer deaths after 30 days and more of the deaths that do occur being attributed to non‐CV causes.^11^, ^12^, ^13^, ^14^ However, the risk of in‐hospital and post‐discharge mortality following NSTEMI is higher in patients with type 2 NSTEMI than in those with type 1 NSTEMI.^20^, ^26^ This is not surprising and can be explained by the sicker and older age demographic of patients with type 2 NSTEMI compared with those with type 1 NSTEMI.^20^, ^33^, ^34^

The objective of current NSTEMI management guidelines is to provide recommendations for the relief of ischemia during hospitalization and, in the longer term, to prevent secondary events such as recurrent MI, events in other vascular beds, and death.^1^, ^3^ All NSTEMI events should initially be diagnosed and managed in accordance with these guidelines regardless of MI type. However, current NSTEMI guidelines are more suited to type 1 NSTEMI than type 2 NSTEMI. Recent guidelines for MI classification have attempted to address this by providing an algorithmic approach to understanding the mechanistic basis for the occurrence of type 2 NSTEMI to facilitate appropriate treatment. Further studies are needed to address this unmet need in the treatment of the older, sicker patient with a type 2 NSTEMI. Meanwhile, given the preponderance of type 1 MI over type 2 MI in patients presenting with NSTEMI, and given the increased intermediate‐ and long‐term risk of major adverse CV events relative to STEMI, aggressive strategies need to be adopted in treating these patients. These include the appropriate use of early invasive approaches, antithrombotic agents that yield the best benefit‐risk outcomes, and other guideline‐directed therapies. On the other side, algorithms that address the multiple possible underlying causes for the myocardial oxygen supply‐demand imbalance in individual patients may be preferable for managing patients with type 2 NSTEMI. Future guidelines should include appropriate evidence‐based recommendations for the management of type 2 NSTEMI.

## CONFLICT OF INTEREST

Marc Cohen has received honoraria as a member of the Speakers Bureau for AstraZeneca.
